# Forms of Disease in Which Opiates Are Indicated

**Published:** 1845-12

**Authors:** 


*Forms of Disease in which Opiates are indicated.*—Dr. Sobernheim furnishes us with the summary of the diseases in which opium is beneficial, and the form of its exhibition:

1. *Fevers Intermittent*, when offering a nervous character, (strong shivering, great anguish, unusual excitement, followed by exhaustion, giddiness, heaviness of the head, hallucinations, and alienation of the senses, spasmodic and convulsive symptoms; pulse small, frequent, and irregular, skin cool, cold extremities, violent vomiting and purging.) In such cases, it is either administered in large doses (from 1 to 3 grains) shortly before the paroxysm, or a little after the shivering has commenced (4 to 6 drops of tinctute every hour, with chamomile tea,) or in the intermission (a quarter of a grain of opium with two grains of quinine every three hours;) if the disease be combined with apoplectic phenomena, venesection is to precede it; if with gastric symptoms, an emetic. Græfe and Luders recommend opium (a quarter of a grain) with quinine (2 grains) against the dangerous shivering, subsequent to deep traumatic lesions, undoubtedly caused by the reflex action of the spinal marrow. Malgaigne also uses opium with the greatest success, after serious operations, to prevent traumatic inflammations. He administers from 6 to 10 grains of opium during the day, and continues it as long as inflammation is to be feared. By this method he asserts to have prevented forever, local inflammation, and even pain. *Typhus fever*, with great excitability, (exhausting discharges, great excitement and sensitiveness, loquacious delirium, continual sleeplessness, pulse small, spasmodically contracted, skin dry, extremities cold,) in small, repeated doses, in the form of *Dover's powder*, with *ammon. carbon. pyro-oleos.*, to encourage the crisis of the skin, but with great caution. In those forms of *typhus abdominalis*, which are combined with inflammation and ulceration of the intestinal mucous membrane, it is to be employed, according to Lesser (most advantageously in the form of clyster,) when the alvine evacuations cause great exhaustion, and appear peculiarly discolored.

2. *Inflammations*, particularly of membranous, glandular, and sensitive organs, assuming a lymphatic character in the second stage, when the inflammatory symptoms are already removed, combined with calomel; against *hepatitis*, with icte rus, after venesection; against *gastritis venenata*, in the form of emulsion, to counteract the violent vomiting; against inflammation of the bladder, (*cystitis*,) with calomel, and under the form of clyster, (P. Frank, Richter;) also against *pneumonia*, when, after antiphlogistic treatment, a state of irritation is left behind, characterised by great excitement, transient, lancinating pains, dry, teazing, spasmodic cough, small, accelerated, and spasmodic pulse, cool skin, &c. In such cases, opium (from a quarter to half a grain,) with small doses of ipecacuanha and camphor (and with calomel, if the inflammation be not yet quite removed,) rendered the most eminent services, according to P. Frank, G. A. Richter, S. G. Vogel, and Hufeland; this particularly refers to *rheumatic* pneumonia and pleuritis. Also against *pneumonia notha* (false inflammation of the lungs,) which is based on a spasmodic affection of the pneumo-gastric nerve, and on a profuse gathering of mucus in the bronchial vessels, (with camphor, ipecacuanha, sulphur auratum, benzoic acid, and senega.) *Feverish exanthemata*, attended by exhaustion, dryness of the skin, if the eruption does not properly come out, or if it seems inclined to recede; particularly in *small pox*, in the stage of eruption and suppuration, (Sydenham, Richter, Reil, P. Frank—*non paucos ex Orci faucibus eripuit*,) with musk, succinate of ammonia, angelica, camphor; against *measles*, with diarrhœa, vomiting, violent cough; with great caution in *scarlatina*, on account of the frequent congestions towards the chest and head.

3. In *rheumatisms*, when without fever, and very painful, arising from suppressed perspiration; combined with ipecacuanha, (Dover's powder,) camphor, and preparations of antimony, it is very advantageous. Lately, opium has also been used successfully (in large doses) against rheumatisms with fever; Cazenave orders pills containing 1 grain of opium, and continues them hourly (!) till the pain ceases, or perspiration ensues. Corrigan and Hope found opium likewise very useful against acute rheumatism. The excellent effect of opium in this disease was proved in one case, where 8 venesections during 12 hours (!) emetics, sal ammonia, nitre, calomel, &c., were all employed in vain, till Benewitz administered the aromatic tincture of opium (rising from *m* xx to ʒss.) with antimonial wine and nitre, and afterwards a quarter of a grain of opium with calomel, ipecacuanha, and sulphur auratum, and experienced the best results. Notwithstanding this success of opium, it is indispensable to bear in mind the following excellent sentence of Wedel: "Sacra vitae anchora est opium bené et circumspecte agentibus, cymba autem Charonis in manu imperiti et seu gladium in manu furiosi."

4. In *gout*, if the paroxysm occasions violent pains; or if it has receded towards the internal organs, opium may help to bring it back towards the skin (combined with ipecacuanha, camphor, antimony.)

5. *Catarrhal* complaints, when chonic and inveterate, with teazing cough and difficult expectoration (with antimony and mercury,) particularly when tending to pulmonary consumption, combined with bitter-sweet, liver of sulphur, &c.

6. *Morbid fluxes.* (a) In *dysentery*, with rheumatic catarrhal character, in harvest time, with little or no fever, opium must be employed as the chief remedy, with small doses of ipecacuanha and calomel; also in the nervous forms, accompanied with exhaustion, &c. Opium is, however, injurious in inflammatory dysentery, with fever or with gastrico bilious complication. (b) *Cholera*, in the sporadic form (from six to twelve drops of the aromatic tincture every quarter of an hour, with some acetic ether;) also frictions on the gastric region, and clysters of opium; in the *epidemic* form, opium was only advantageously used against the lighter cases; whilst in the *asphyxiated* or *pulseless* modification, opium was even injurious, by the untimely stoppage of the secretions, as was shown in the late epidemic of cholera at Berlin (Autumn, 1837.) (c) *Diabetes* (with animal diet;) Bailie gives it with rhubarb; Berndt with ammoniuretted copper. (d) *Diarrhœa*, not caused by inflammatory action, particularly of a gastrico-rheumatic character, if very frothy, watery, and exhausting; also with great care during the period of dentition. C. Vogel recommends it against the diarrhœa, sleeplessness, emaciation, weakness, and convulsions, which generally precede softening of the stomach; Cruveilhier, against gastritic complaints, characterized by a peculiar state of the stool, resembling froth of eggs. (e) *Emesis* (vomiting,) purely spasmodic and nervous. (f) *Hemorrhages*, particularly of the lungs, and hæmoptysis (spitting of blood,) with ipecacuanha (Jahn says, "I do not hesitate to declare opium the most indispensable remedy in this disease;") and in spasmodic uterine hemorrhages (with tincture of cinnamon, elixir of vitriol, &c.)

7. *Morbid retentions*, by spasmodic contraction of the vessels, and of the eductory canals. (a) The *icterus* of irritable, hypochondriacal individuals, after cold or mental excitement. (b) *Dropsy*, in consequence of receded eruptions, particularly anasarca and thoracic dropsy, combined with foxglove and calomel; also as an excellent corrective with squill and other violent diuretics. (c) In *painter's colic* it is the chief remedy, either by itself or with alum; sometimes with calomel, castor oil, or in a solution of sulphate of soda (Richter;) here it quickly removes the spasmodic obstruction. (d) In *spasmodic and flatulent colic* (with aromatics and naphtha). (e) In *retention of urine*, when spasmodic; also used in external frictions on the region of the bladder, together with warm oil of chamomile, and in clysters.

8. In *spasms and pains*, when caused by derangement of the primary nervous functions, particularly by morbid increase of the sensitive nervous actions, and comparative inaction of the irritable system, opium restores the original equilibrium, and is thus the chief remedy. Cullen particularly advocates it in his so-called "nervous lumbago," after previous local bleeding.

9. *Neuroses*, viz.—(a) *hooping-cough*, but only in the second or nervous stage; (b) *hydrophobia*, in large doses, as a preservative, after venesection has been carried to fainting; (e) in *trismus and tetanus* it is a chief remedy, particularly when they are traumatic or rheumatic, (Stütz gave it with carbonate of potash and potash baths,) in increasing doses, to begin with two grains, and increasing a quarter of a grain every hour; or twenty drops of the tincture, and to rise five drops every hour, (in symptoms of poisoning black coffee is to be given along with it, and then again twenty drops of the tincture to be continued, Rust); in tetanus, proceeding from gangrena senilis, it is particularly praised by Wendt; (d) in *delirium tremens*, in large doses (from one to twenty grains every hour, till critical sleep appears; if complicated with plethora of the head, hard full pulse, and suppression of the usual secretions, venesection is to precede it; if with gastric symptoms, an emetic, particularly tartrate of antimony and potash; also in *madness*, not based on organic changes, but on suppressed perspiration, sexual excitement, &c. (then to be combined with camphor;) in spasmodic phenomena, fright, &c.

10. In *pulmonary phthsis*, with tormenting cough, profuse expectoration, and great sensibility; in the night perspirations opium is to be combined with acetate of lead.

11. In *gangrene*, with characters of debility, particularly in gangr. senilis and hospital gangrene, internally and externally.

12. In *syphilis*, with painful and spasmodic symptoms, (an important corrective with mercurial preparations,) particularly in nightly pains of the bones (in the form of acetate of morphia.)

13. In *poisoning by caustic metallic oxides*, if, after the use of proper antidotes, a state of heightened irritability is left in the nervous system; also against poisoning by remedies of very acrid principles (as veratrum album, helleborus, squill, colchicum, semen sabadil.)

14. *Externally*, (a) in all painful and spasmodic maladies, particularly spasmodic colic, pain through calculi (with warm chamomile oil,) painful erysipelatous affections, cancers, phymosis and paraphymosis, chordee, irritable gonorrhœa, leucorrhœa, and painful ulcers; (b) in some *eye complaints* it is a chief remedy, particularly in chronic ophthalmia left after the acute inflammation, when great sensibility, lachrymation, photophobia, and spasms of the eyelids remain behind; and particularly in arthritic, syphilitic, blenorrhagic and metastatic ophthalmia; against varices of the conjunctiva, obscurations of the cornea (Lallemand,) ulceration, pannus, pterygium, staphyloma; it is also an excellent remedy in polypus of the nose, (the polypi are to be daily touched with laudanum, and covered with lint imbued in the same tincture,) polypi of the ear and uterus; *chilblain*, frost-bitten limbs.—*Ibid.*

